# Equitable access to quality trauma systems in Ghana: a qualitative study

**DOI:** 10.1136/bmjopen-2024-087095

**Published:** 2025-02-12

**Authors:** Sheba Mary Pognaa Kunfah, Mustapha Yakubu, Bernard Ofori-Appiah, Emmanuel Ayingayure, Maria Lisa Odland, Tolgou Yempabe, Alexis Dun Bo-ib Buunaaim, Dominic Konadu-Yeboah, Agnieszka Ignatowicz, Justine Davies, Tabiri Stephen

**Affiliations:** 1 Department of Public Health, Tamale Teaching Hospital, Tamale, Northern Region, Ghana; 2 School of Medicine and Health Sciences, University for Development Studies School of Medicine, Tamale, Northern Region, Ghana; 3 Global Surgery Unit, National Institute for Health and Care Research, Tamale, Northern Region, Ghana; 4 Department of Surgery, Tamale Teaching Hospital, Tamale, Northern Region, Ghana; 5 Institute of Applied Health Research, University of Birmingham, Birmingham, UK; 6 Department of Obstetrics and Gynecology, St. Olav’s University Hospital, Norway, UK; 7 Department of Surgery, Trauma and Orthopedics, Tamale Teaching Hospital, Tamale, Northern Region, Ghana; 8 Kwame Nkrumah University of Science and Technology School of Medical Sciences, Kumasi, Ashanti, Ghana; 9 Komfo Anokye Teaching Hospital, Kumasi, Ashanti, Ghana; 10 University of Birmingham, Birmingham, UK; 11 Global Health, University of Birmingham, Birmingham, UK; 12 School of Medicine, University for Development Studies, Tamale, Ghana

**Keywords:** TRAUMA MANAGEMENT, PUBLIC HEALTH, Health Equity, Health Services Accessibility

## Abstract

**Abstract:**

**Objectives:**

To explore the barriers to accessing quality trauma care after injury in Ghana.

**Design:**

A qualitative study using semi-structured interviews and focus group discussions in one rural and one urban setting. Interviews and focus group discussions were audio recorded, transcribed and thematically analysed using the four-delay framework.

**Participants:**

53 patient participants (n=39 men, n=14 women, mean age=41, SD=15.6, n=38 rural participants, n=15 urban participants) who had an injury not more than 6 months preceding the start of the study.

**Settings:**

15 individual interviews (n=15) and 2 focus group discussions (n=23) were conducted in Yendi (rural setting in Ghana) and 10 individual interviews (n=10) and 1 focus group discussion (n=5) in the Tamale metropolis (urban setting in Ghana).

**Results:**

Our findings showed that when an injury occurred, participants faced multiple barriers across all delays which prevented them from accessing quality injury care. Barriers were a mix of individual, community-level and health-system factors that were interrelated in many ways. Financial difficulties were one of the prominent barriers mentioned by the participants in both settings.

**Conclusion:**

This study shows that multiple factors cause an injured patient to delay in seeking care, reaching care, receiving care and remaining in care. Therefore, there is a pressing need for comprehensive, community-driven strategies to strengthen health literacy at the community level. There is also a need for facility-based strategies that would improve the availability of medical and human resources to augment access to quality trauma care. Additionally, if policymakers focus on removing financial barriers to trauma care and strengthening referral systems, especially in the remote and rural areas, it would greatly improve access to quality trauma care in Ghana.

**STRENGTHS AND LIMITATIONS OF THIS STUDY**

- Combining individual interviews and focus groups enhanced the depth, breadth and validity of our study for a richer and more nuanced understanding of the topic.
- This study would have been more representative of the country if the data collection had been expanded to cover other regions, particularly the southern part.
- Including the perspective of healthcare professionals would have further enriched the findings of this study.
- The majority of the participants in the study had injuries from road traffic accidents, and hence this may affect the generalisability of the results to other injury types.
- There were more participants from the rural setting than from the urban setting, which may have led to more barriers recorded from the rural setting.

## Introduction

 Injuries pose a significant challenge to individuals, communities, nations and the world at large. According to the WHO, each year, nearly 4.4 million lives are lost as a result of injuries, representing 8% of all global mortalities.^1^ It has also been shown that nearly 90% of these injuries occur in low- or middle-income countries (LMICs).^1^ The economic burden of injuries is significantly high, and millions of dollars could be saved with appropriate interventions. Injury also reduces the quality of life for survivors, with disabilities placing considerable and enduring strains on both patients and healthcare providers.^1^,^3^

Over the last three decades, injuries and deaths from road traffic accidents have increased.^4^ According to the Ghana Road Safety Commission, the first half of 2024 alone saw nearly a 10% increase in road trafficaccident-related deaths.^5^ A study conducted in a district in Ghana showed that about 64% of commercial motorcyclists had been involved in a road traffic accident at least once.^6^ In a major hospital in Ghana, over 2 years (2016 and 2017), about 17 000 injuries were reported to the emergency room.^6^ Similarly, another study in Ghana found that 50% of injuries were from falls, and 25% were due to road traffic accidents.^7^

While stakeholders focus on preventive measures to reduce road traffic accidents, injuries remain inevitable. Hence, it is prudent to refocus efforts on improving access to quality trauma care. However, in Rwanda, for instance, researchers found persistent delays at different stages of the trauma care pathway.^8^ Identifying these barriers can help address issues and improve survival and post-trauma outcomes.^9^ In some LMICs, studies have highlighted complex, context-specific barriers, such as limited prehospital services, insufficient hospital resources and financial constraints, that delay access to quality trauma care.^810^,^13^ For instance, in South Africa, researchers found a 60% reduction in trauma-related deaths by minimising delays to trauma care through improvements in health systems.^14^ Despite these studies showing the need to redirect research on trauma care, most studies in Ghana have focused on the epidemiology and prevalence of injuries, leaving gaps in understanding the full trauma care pathway.^6 7 15^

The three-delay framework, originally developed to address maternal mortality, evaluates barriers related to seeking care (delay 1), reaching care (delay 2) and receiving appropriate care (delay 3).^16^ Although it has been widely used in evaluating maternal and child health systems,^17^,^19^ very few studies in emergency and trauma care systems have used this model.^12 14 20^ A systematic review found that only 2.7% of trauma studies in LMICs fully used the three-delay model, with nearly 80% focusing solely on delay 3.^21^ Another study on barriers to quality care in Ghana examined only delay 3, revealing that cost was a primary barrier, but no comprehensive exploration of all delays has been conducted.^22^

In recent years, a fourth delay, remaining in care (delay 4), has been introduced, providing insights into follow-up care to ensure a holistic understanding of the trauma care pathway, including decisions to seek, reach, receive and remain in care.^10 13^ Though studies have examined individual barriers to care in Ghana, none have applied the four-delay framework to assess equitable access to trauma care. Our study, therefore, aims to fill this gap by using a qualitative approach to evaluate barriers to trauma care in Ghana through the four-delay framework. This work will also direct future research and inform policy changes to develop more equitable and effective trauma care systems in the country.

## Research methods

### Study setting

This study was conducted in the Northern Region of Ghana as part of a larger project that sought to explore access to care after injury in three LMICs (Equi-Trauma). Adibo and Bumbongnaayili in the Yendi district (rural settings) and Tamale Metropolis (urban setting) were the chosen sites. Researchers considered the diversity in healthcare systems and the cultural and economic diversities in selecting these sites to ensure a full representation of the general population. The Northern Region of Ghana is the fifth largest region, with a population of about 2 310 939, with 52.6% being rural. Tamale Metropolis is the capital and is mainly urban, with a population of about 374 744. The Yendi district is mainly rural, with a population of around 154 421. The most widely spoken language in the region is Dagbanli, followed by Gonja.^23^

### Sampling and data collection

The data collection period was from October 2020 to January 2021. Purposive and convenience sampling was used to recruit participants for the study. Participants were included if they had accessed healthcare for an injury within the last 6 months preceding the study, were 18 years and older and were able to provide informed consent. The recruitment criteria were also based on participant availability and willingness during the data collection period. Patients were excluded if they were still receiving inpatient care and were not willing or able to provide consent to participate in the study.

In the urban setting, a community member who was familiar with the community was recruited to assist two trained research assistants to identify eligible participants. In the rural setting, health workers in health centres reviewed patient records to identify the eligible participants together with the research assistants.

Participants were contacted by phone, and with the assistance of a recruited community member, they were traced to their homes to share information about the study using participant information sheets. After about 1–2 weeks, these participants were contacted to assess their willingness to be part of the study and to book a convenient location for the data collection. If they were willing to be part of the study, they were asked to sign or thumbprint a consent form before data collection took place.

Focus group discussions (FGDs) were held, one in the urban and two in the rural settings (one in each rural community). The two focus group discussions conducted in the Yendi district were to give a complete representation of the cultural and economic diversities present in the two different communities selected. All focus group discussions had groups of 6–12 participants with a total number of 28 participants. There were 25 individual interviews conducted in total, with 10 in the Tamale Metropolis and 15 in the Yendi district. Each participant gave informed consent before data collection. Trained research assistants used interview and focus group discussion guides to lead the discussions (see online supplemental file 1). The interviews and focus group discussions lasted 1–2 hours and were audio recorded using a dictaphone.

### Data analysis

Interviews and FGDs were audio-recorded in both English and Dagbanli (the local language of the research sites), depending on the preference of respondents. The interviews in Dagbanli were translated to English by the research assistants (EA and MY) and reviewed by one of the researchers who did not participate in the interviews to ensure translation accuracy (A-MA-L). Interviews and focus group discussions were transcribed by the research assistants (SMPK and MY). All the transcripts were cross-checked by four researchers who did not participate in the interviews (TS, TY, ADB-iB and DK-Y). Two research authors (SMPK and MY) initially coded each transcript, which was later cross-validated by other participating researchers (AI, BO-A, EA and AM). Coding was conducted with a thematic analysis approach using the four-delay framework seeking care (from the time of injury to the time the decision was made to seek care), reaching care (the time the decision was made to seek care to the point where they got to the first healthcare facility), receiving care (the time where they got to the health facility to the time they were discharged from the health facilities) and remaining in care (the time between discharge from a health facility to the end of review visits). This framework formed the broad themes for the analysis of the data. All discrepancies were resolved through several discussions held weekly throughout the data collection and analysis period. Researchers also maintained objectivity, collaboration and cultural sensitivity during data collection and analysis. They constantly acknowledged their personal biases to ensure validity and transparency in the study.

### Public and patient involvement statement

Community leaders and members were consulted before the project to gain insights into their cultural contexts, obtain approval to carry out the research in their communities, understand local recruitment strategies and receive support for the recruitment of participants. A workshop was also organised with key community leaders and health workers to validate the study’s findings.

## Results

**Figure 1:**
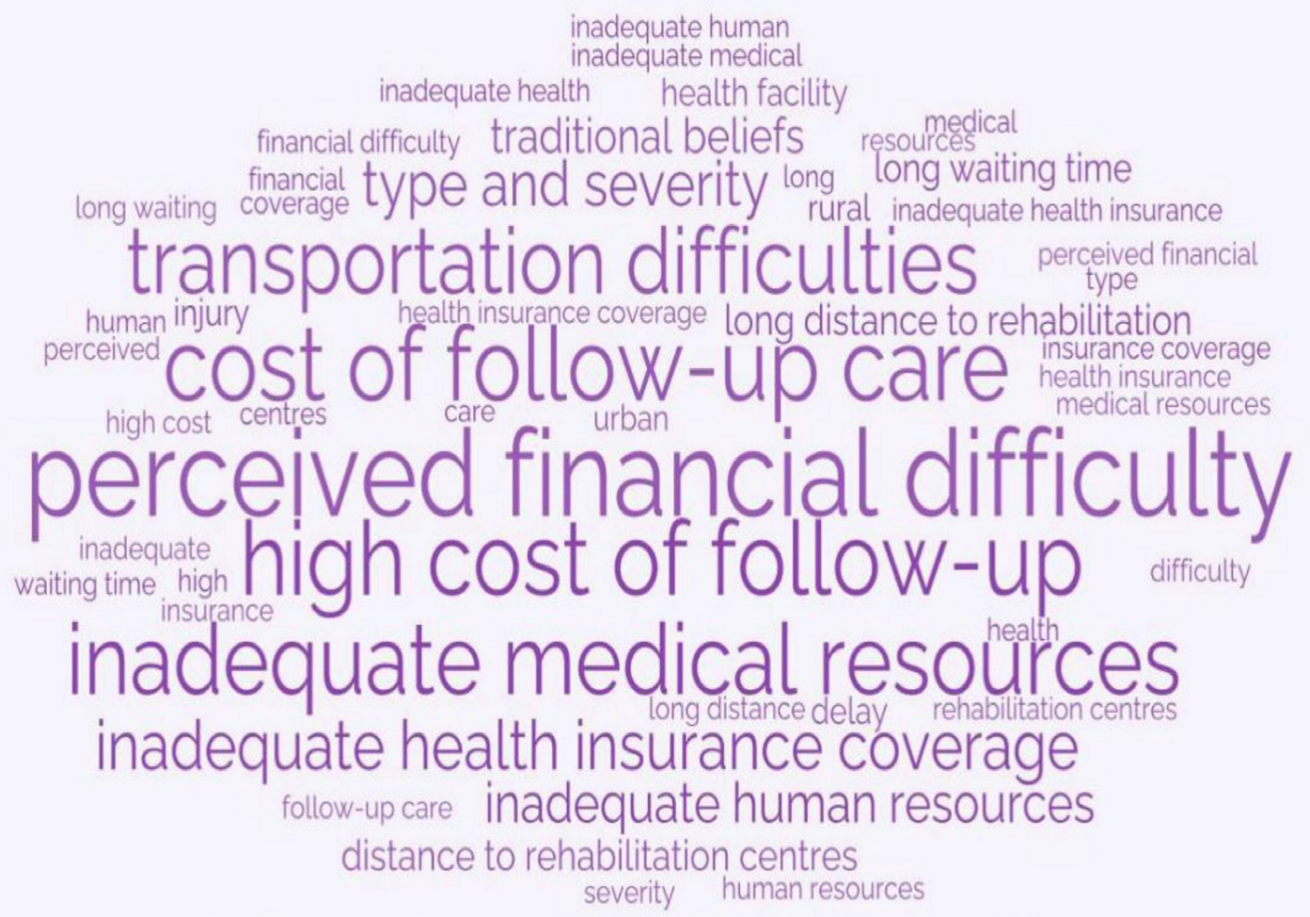
A word cloud highlighting the various barriers to trauma care in Ghana. Large text—most frequently occurring, medium-sized text—moderately occurring and small text—least occurring.

Generally, the barriers mentioned were similar in the rural and urban settings. However, out of a total of 213 barriers, the rural setting reported more barriers than the urban setting. The most common barrier mentioned throughout the study in both study settings was financial difficulties, which was reported as perceived financial difficulties in delay 1 and high cost of follow-up care in delay 4, as shown in table 1. Figure 1 shows a word cloud of all the barriers reported in this study.

**Table 1:** List of delays, barriers and their frequency of occurrence

Delays	Ruraln (%)	Urbann (%)	Totaln (%)
Delay 1			
Perceived financial difficulty	20 (9.7)	20 (9.7)	40 (19.3)
Health illiteracy	14 (6.8)	5 (2.4)	19 (9.2)
Traditional beliefs	6 (2.9)	5 (2.4)	11 (5.3)
Delay 2			
Transportation difficulties	28 (13.6)	10 (4.8)	38 (18.4)
Distance to a health facility	3 (1.4)	1 (0.5)	4 (1.9)
Delay 3			
Inadequate medical resources	20 (9.7)	10 (4.8)	30 (14.5)
Understaffing of health facilities	10 (4.8)	1 (0.5)	11 (5.3)
Inadequate health insurance coverage	5 (2.4)	8 (3.9)	13 (6.3)
Delay 4			
High cost of follow-up care	23 (11.1)	9 (4.3)	32 (15.5)
Long distance to rehabilitation centres	3 (1.4)	2 (1)	5 (2.4)
Long waiting time	1 (0.5)	3 (1.4)	4 (1.9)
Overall total	133 (64.3)	74 (35.7)	207 (100)

### Demographic characteristics of participants

The demographic characteristics of the participants are presented in table 2. The majority of the participants were men (80%). The mean age was 41±15.56 years, and most of the injuries were from road traffic accidents.

**Table 2:** Demographic characteristics of participants in individual interviews and focus group discussions (FGDs) (n=53)

Average age (SD) 41 (15.56)			
Variable	Type	Participants (individual interviews)	Participants (FGD)
Sex	Male	20	19
	Female	5	9
Area of living	Rural	15	23
	Urban	10	5
Language used	English	10	0
	Dagbani	15	28
Mechanisms of injury	Road traffic accidents	20	22
	Others*	5	6
Types of injury	Limb fractures	14	17
	Head injury	1	2
	Soft tissue injury	8	7
	Dislocations	2	2

* Fall from heights, bee stings and blunt injury.

### Delay in seeking care (delay 1)

When an injury occurred, several factors delayed the decision to seek care. The perceived financial difficulties, health illiteracy and traditional beliefs were the most commonly reported barriers causing a delay in deciding to seek care.

#### Perceived financial difficulties

Most participants mentioned the perceived cost of treatment in healthcare facilities and the cost of transportation to a place of care as hindrances to seeking care. The patients and their families would often first assess their financial status. If they had inadequate resources to meet the perceived cost of care, they would either wait at home until they could raise some funds or they would decide not to seek care. One of the participants recounted the story of a boy who fell off a tree, and his family delayed seeking care because they could not raise adequate funds, and this resulted in severe complications.

Some years back, one boy in my school climbed a tree, and his leg got broken so the family was not having anything, so they left the boy in the house. They couldn’t send him to the herbalist or the hospital for three or 4 days until the leg started rotting (…). Because the family didn’t have anything… Patient Participant 005, Urban

It wasn’t anything else but lack of money. Patient Participant 001, Rural

#### Health illiteracy

When an accident occurs, the patient or the bystanders usually assess the type and severity of the injury. If the patient had no obvious wounds, was conscious or had wounds believed to be minor, the decision to seek care would be delayed, often until the condition worsened.

Our people don’t believe that someone can get an injury or trauma, be conscious at that moment and later develop complications. So far as the person is conscious at the scene of the accident, it is believed that the injury can be managed at home. FGD Patient Participant 005, Rural

And if the injuries are not severe or not bleeding but is a fracture, we don’t go to the hospital. FGD Patient Participant 013, Urban

#### Traditional beliefs

Participants mentioned a traditional belief that the decision to seek care was the sole responsibility of the family head. Therefore, if the family head was not around, the injured person had to wait at home until the head was consulted.

…I had an accident, then people came and took me to my uncle’s house (…). My uncle was not there. I was there [in the uncle’s house] for one (1) day until my uncle arrived, and then [my uncle decided to] send me to [name] hospital. FGD Patient Participant 016, Urban

### Delay in reaching care (delay 2)

The barriers that contributed to the delay in reaching care were transportation difficulties and distance to health facilities. These two barriers affected rural and urban communities in different ways.

#### Transportation difficulties

Most participants said they had bad roads, especially in the rainy season, and this problem was worsened by the lack of ambulance services in their communities. Sometimes they had to improvise with alternative sources of transportation such as tricycles or motorbikes.

If you have to go to Naya [town], you can sit on the road for a long time and still not get a vehicle to go there. Patient Participant 003, Rural

Here we don’t have an ambulance, so when someone gets an injury and can sit on a motorbike, we use the motorbike to send the person. However, if the person cannot sit on a motorbike, we’d look for a tricycle or hire a car. Because we don’t have an ambulance. FGD Patient Participant 007, Rural

#### Distance to a health facility

The distance to health facilities was also a common barrier that affected rural communities more than urban communities. Unlike the urban community, the rural communities did not have nearby health facilities. Therefore, when an injury occurred, they had to travel long distances to the next community or town with a health facility for care.

(…) 3 to 4 hours, if we ride faster [to a hospital], we can use 3 hours, if slowly 4 hours. Patient Participant 008, Rural

### Delay in receiving care (delay 3)

Participants detailed various barriers that caused delays in receiving quality care when the injured person reached a healthcare facility. Barriers such as understaffing of health facilities were predominant in rural settings than urban ones. The common barriers mentioned by participants in both rural and urban communities included inadequate health insurance coverage and inadequate medical resources.

#### Inadequate health insurance coverage

The National Health Insurance does not fully cover trauma care leading to high out-of-pocket payments. The high rate of poverty in this study area further compounded the patients’ inability to afford quality trauma care, thus delaying treatment. This caused some patients to leave hospitals to seek care from traditional healers.

I was made to understand that the health insurance does not cover that… so I had to pay for the computer tomography (CT) scan. Patient Participant 004, Urban

(…) the surgery honestly, the amount of money they [healthcare workers] mentioned wasn’t something we could afford (…) that’s why I decided to go for local treatment. Patient Participant 003, Urban

#### Inadequate medical resources

Participants mentioned that most health facilities lacked medications which meant that patients had to purchase items like medicines and dressings from outside the hospital. In some situations where medical equipment or medication was not available, patients had to access such services from private enterprises (pharmacies, laboratories, etc) outside the hospital, which caused a delay in receiving care.

(…) and we faced some challenges with the CT scan. (…). The CT scan was broken at the hospital. So, they [healthcare workers] even recommended that we should go to Techiman or is it Sunyani [4 hours’ drive from Tamale] to do the scan. Patient Participant 004, Urban

#### Understaffing of health facilities

The health workers serving the rural areas are inadequate and therefore are unable to provide 24-hour services, thus patients had to wait until the staff were on duty to address their needs.

Concerning accidents, most of the nurses who can dress wounds and attend to you properly when you are involved in an accident do not stay in the town, (…) so if the accident occurs at night when they have all gone home, then you would be in serious trouble. Rural Patient Participant 004, Rural

### Delay in remaining in care (delay 4)

Although most participants knew that they were to return to the health facility for various purposes ranging from follow-up reviews and wound dressing to physiotherapy, factors such as long distances to rehabilitation centres, long waiting times and the high cost of follow-up care were barriers contributing to the delay in remaining in care.

#### Long distances to rehabilitation centres

The participants mentioned that most health facilities lacked rehabilitation services, hence the need to travel long distances to access such services. This challenge made it difficult for the patient to comply with the follow-up schedules.

it is very far because from here to [big town] will not be a short journey. Patient Participant 007, Rural

…distance to the hospital might hinder hospital revisits for reviews. Patient Participant 004, Urban

#### Long waiting time

Participants stated that long queues at hospitals were a major factor resulting in non-compliance to review visits. Some mentioned that they could be in a queue to see a doctor only to be disappointed and asked to return on a different day.

(…) the queue will be too long and according to the doctors and the nurses; they would attend to only 30 patients. The rest of you should go home (…). FDG Patient Participant 014, Urban

(…) the number of times I’ve been there it has always been hell (…) and even those days that we have been there, you realise that the place is full. Patient Participant 004, Urban

#### High cost of follow-up care

The cost involved in transportation, medications and rehabilitation services prevented some patients from remaining in care. Participants also said the cost of receiving care impacted them financially, and they could no longer afford the additional follow-up cost.

Well, in most cases, it is because they have spent a lot of money already at the hospital (…) When the day finally comes and he/she doesn’t have money, he/she isn’t able to go back. Patient Participant 008, Urban

Sometimes as you go back for the wound dressing, you might have to pay money, and because you don’t have money, you have to stay at home and treat it yourself. FGD Patient Participants 0023, Rural

## Discussion

To the best of 0ur knowledge, this study is the first to explore barriers to accessing quality trauma care after injury in Ghana using the four-delay framework. The study showed that when an injury occurred, patients were faced with multiple barriers that prevented them from accessing timely and quality trauma care. Generally, these barriers were a combination of individual, community and health system factors with a degree of interrelatedness.

Financial difficulties as a barrier was frequently mentioned as a major delay in seeking care. The Northern Region is reported to have the highest number of poor people in Ghana.^23^ It is, therefore, not surprising that financial difficulties was a major barrier to seeking care, especially in the rural setting. This is similar to a study conducted in Ghana on the barriers to acute stroke care, where it was highlighted that patients or their caregivers were delayed in seeking healthcare due to financial constraints.^24^ This barrier was also the most frequently mentioned in the entire study. Therefore, if policymakers target interventions aimed at removing financial barriers to trauma care, it would improve access in a significant way.

Health illiteracy also seemed to be a prominent barrier. The participants considered the consciousness of the patient and injuries that did not have any obvious bleeding as mild and not requiring medical attention, which may be an incorrect clinical assessment of their injury. This finding is similar to an interpretive synthesis of studies conducted in Ghana, where it was found that breast cancer victims delayed in seeking care due to the misinterpretation of some of their symptoms as not requiring medical attention.^25^ This poor health literacy was also seen in a systematic review of studies conducted in low- and middle-income countries that looked at the barriers to accessing out-of-hospital care. It was identified that the inability of the patient to recognise danger signs and the severity of their condition was a major barrier to seeking health care.^26^ Health literacy through community education should be emphasised to improve health-seeking behaviour.

Socio-cultural and traditional beliefs were also barriers that prevented patients from seeking timely healthcare. In our study, the family head was most likely the final decision maker in the area of seeking care. Therefore, when the family head was not available, the injured faced a delay in seeking care. This situation is similar to a study conducted in some rural communities in Ghana, where pregnant women had no autonomy in decision-making in healthcare. Even with emergency complications, they had to wait for their spouses or other family members to permit them to seek health care.^27^ There is therefore a need for further studies on the impact of gender norms and culture on health-seeking behaviour within the Ghanaian context.

In our study, geographical access to a health facility was a common barrier, especially for patients in rural areas. In the rural settings, most patients travelled about 17 km to access primary healthcare. This finding in our study has also been highlighted in a study conducted in rural communities in Ghana, where several geographical barriers to healthcare exist in the context of maternal health. Some patients had to cover distances as much as 20–40 km, which meant travelling several hours to the nearest health facility.^28^ This study provides evidence to support the need to improve primary healthcare which would also contribute greatly to the goal of attaining Universal Health Coverage.

Our findings show that the lack of medical equipment like CT scans causes a delay in receiving adequate healthcare for trauma patients. This is similar to another study conducted in Ghana which highlighted that the lack of proper equipment for diagnostic imaging and surgical procedures hinders the prompt and appropriate management of trauma patients leading to potential complications and poor patient outcomes.^29^ The understaffing of health facilities, especially in rural areas, is a barrier to timely trauma care. Patients visit facilities and health staff are not available to attend to them even in emergency settings. This situation is similar in higher-level facilities such as hospitals, where there are inadequate trauma surgeons, emergency physicians and nurses, which also affects their ability to deliver timely and specialised care to trauma patients.^29^ Additionally, high out-of-pocket expenditure associated with inadequate health insurance coverage results in suboptimal in-hospital treatment.^29^ This is in line with our study which highlights that lack of insurance coverage for the cost of trauma surgery in particular is a major cause of delay in receiving care. The National Health Insurance Scheme is part of the strategy to reduce high out-of-pocket expenditures. However, this scheme is faced with a lot of challenges, hence impacting negatively on the health-seeking behaviour of the people, especially the poor.^30 31^ Patients rather opt for traditional healing which is perceived to be relatively cheaper compared with formal healthcare, as shown in a study carried out in the Northern Region of Ghana.^32^ To address these barriers, strategies such as improving healthcare infrastructure, especially in remote communities, increasing the number of trained healthcare professionals, addressing the shortage of essential medical supplies and equipment and implementing health insurance schemes targeted at trauma patients to ensure that cost is not a barrier to accessing timely and appropriate care.

In Northern Ghana, there are inadequate physiotherapy centres, which are limited to the capital town, meaning that patients from remote communities are required to travel long distances to access this service. It was, therefore, not surprising that the patients in this study mentioned long distances to rehabilitation services as one of the barriers to remaining in care. A study conducted in some facilities in Ghana highlighted that out of nine regional and district facilities assessed, only one of them had inpatient rehabilitation services. Five out of the nine facilities did not have a physiotherapy gymnasium, and sometimes patients had to travel an average of 22 km to access rehabilitation services.^33^ Policymakers in Ghana and other LMICs need to consider exploring cost-effective ways of providing such services. Implementing physiotherapy outreach services could go a long way to improve access to rehabilitation services. Long hospital queues and, in effect, long waiting hours were also noted to be a contributing factor to patients’ hesitancy to return for reviews and rehabilitation. This barrier was also found among stroke patients who accessed rehabilitation services in Ghana.^34^ When patients wait for long hours at healthcare facilities, this negative experience does not motivate them to remain in care.^35^ Therefore, there is a need for further research to thoroughly identify and prioritise the barriers and facilitators to remaining in care after injury in Ghana.

### Strengths and limitations

This study is the first to employ the four-delay framework to evaluate trauma care in Ghana, significantly contributing to the expanding body of literature on barriers to accessing quality trauma services. The combination of individual interviews and focus groups was to enhance the depth, breadth and validity of our study for a richer and more nuanced understanding of the topic.

Our study had a mixture of personal experiences which may have been difficult to share in a group and shared community-level experiences which were built on other people’s narrations. This allowed participants who may not otherwise have had a platform to voice their challenges following injury to share their experiences. The insights gained from this study highlighted critical gaps that stakeholders must address when developing or implementing policies aimed at improving access to quality trauma care in Ghana. Additionally, this research serves as a valuable reference for future qualitative and quantitative studies focused on trauma systems in the country.

However, our study does have limitations. Given Ghana’s cultural and economic diversity, the findings may not fully represent the experiences of all regions, especially the southern part of the country. Incorporating the perspectives of healthcare professionals could have further enriched the findings and provided a more comprehensive understanding of the trauma care landscape. Furthermore, despite attempts to include participants with different injury mechanisms and from both sexes, the preponderance of road traffic crashes as a cause of injuries and that most injury sufferers are men was reflected in our participant characteristics. The majority of participants interviewed had sustained injuries from road traffic accidents, which may limit the generalisability of the results to other types of injuries. This study also has an underrepresentation of women, especially in the rural area, which may not have amplified the nuanced experiences of the rural woman.

### Conclusion

This study identified several critical barriers to quality trauma care within the framework of the four-delay model, revealing a complex interplay of individual, community and health system factors across rural and urban contexts. Financial constraints, transportation difficulties, resource constraints at the health facility level, inadequate health insurance coverage, cultural beliefs and health illiteracy were among the many barriers identified across rural and urban contexts. Therefore, there is a need for holistic strategies which are community-based to improve the health literacy of people in communities. There is also the need for facility-based strategies that would target improving the availability of medical and human resources to improve access to quality trauma care. Additionally, if policymakers focus on removing financial barriers to trauma care and strengthening referral systems, especially in the remote and rural areas, it would greatly improve access to quality trauma care in Ghana. Longitudinal studies are needed to assess the health outcomes of trauma patients. There is also a need for an evaluation of the current policies on trauma care to assess the policy gaps hindering access to quality trauma care in Ghana.

## supplementary material

10.1136/bmjopen-2024-087095 online supplemental file 1

## Data Availability

Data are available upon reasonable request.
