# Supplementary material for: Equitable access to quality trauma systems in Ghana: a qualitative study
Source: BMJ Open. 2025 Feb 12;15(2):e087095. doi: 10.1136/bmjopen-2024-087095 (PMC11822442; doi:10.1136/bmjopen-2024-087095)
Supplement: online supplemental file 1 [file bmjopen-15-2-s001.doc]

**Supplementary file**

**Equitable access to quality trauma systems in Lower and Middle Income Countries. Assessing gaps and developing priorities**

**Service User In-Depth Interview Topic Guide** Version 1. 25-01-2020 (note this is a discussion guide, the research assistants running the study will be fluent in English and local languages, and will translate discussion points, where needed, for participants)

SWITCH THE DICTAPHONE ON

I realise that you have had an injury in the last 6 months, could you tell me about what happened?

Probe:

Where were you when the injury happened?

How badly were you injured?

How about after your injury happened, what happened?

Probe:

Was anyone there to help? Who was there to help you?

Did you think that you needed medical help? (If not, why not?)

Did you get help at the time incident?

How long did it take for you to get help?

Were there any things in particular that stopped you trying to get help?

Were there any things in particular that delayed you getting medical help?

Probe:

No one there to help

Ambulance (taxi/motor/bicycle) availability?

Road conditions?

Personal safety?

Costs?

Where did you first go to for treatment?

Probe:

Why that particular place? Was it a clinic or a hospital?

How far away was it?

How long did it take you to get there?

How did you get there?

Did you have any problems challenge getting there?

How quickly were you seen?

Do you think that the doctors and nurses had all that they needed to treat your injury well?

How was your care there? Why do you think it was good/bad/OK?

How would you describe the treatment you received from the health facility?

Were you sent somewhere else for care? Why?

Was that the only place you went to, or did you need to (or were you referred to) another place to get care?

Probe:

How did you feel when you received the news?

Did you go to the referred health facility?

How did you get to there?

Note to interviewer: please explore how many places the person needed to go to in order to get definitive care and what happened in those place.

For each place the participant attended, you may want to ask:

How quickly were you seen?

How were you treated? Why?

Did the staff explain things to you?

Did you have to wait for long before getting treatment?

Do you think that the doctors and nurses had all that they needed to treat your injury well?

How long did you stay there?

How did you feel?

Why do you think the doctors sent you somewhere else for care?

What factors do you think would make it more likely for you to reach the right place of care where your injuries would be treated more quickly?

Probe:

For example:

- Ambulance availability?
- Road conditions?
- Costs?

Was there anything in particular that stopped you getting the right treatment?

Probe:

Family commitments meant had to shorten stay?

Did you have to pay for care? Did you have to borrow or sell anything to pay for care?

Too far for relatives to travel to visit?

After your injury was treated, what happened then?

Probe:

Did you go home? Did you stay in the hospital?

How did you feel about your injury and care you received?

When you were discharged, were you asked to go back to see the healthcare workers about your injury?

Probe:

Doctors in a clinical appointment?

Physiotherapists for rehabilitation?

If so, how far did you have to travel to get to these appointments?

Why do you think you were asked to go back? Why not?

Did you manage to keep all of the appointments?

Probe:

Were there any things in particular that you felt limited your ability to keep all these appointments? (E.g. Travel time? Costs? Other commitments? Thought there was no need?)

How are you feeling now? Are you now back to your full fitness?

What do you think could have been different about your care?

Probe:

Do you think that you received an adequate care? Why?

Geographic/transport issues?

Health facility issues?

Thinking about your family, friends or community, what usually happens in Ghana when people get injured?

Probe:

Is your experience similar to other people?

If no, how does it differ?

What happens for people who are discharged from hospital or when their injury has been mostly treated?

Do most people who have been asked to return to care (or remain in care) actually do so? Why?

What do you think are the barriers to people remaining in care?

What are the facilitators to people remaining in care?

Prompts, if necessary:

For example:

Distances needed to travel?

Other family commitments?

Costs?

Transportation challenges?

Finally, what do you think could be done to improve access to quality care for people who are injured in your area?

That was my last question, thank you for telling us about your experiences. Is there anything you would like to add?

Thank you again for your time.

SWITCH THE DICTAPHONE OFF

**Service Users Focus Group Discussion Guide Version 1. 25-01-2020** (note this is a discussion guide, the research assistants running the study will be fluent in English and local languages, and will translate discussion points, where needed, for participants)

**SWITCH THE DICTAPHONE ON**

First, I would like to thank you for attending this discussion. The recordings will be anonymous, and we don’t want you to say your name at the start, but we have given each of you a pseudonym (made up name). We will go around the table in turn asking you to say your pseudonym, what age you are, the area in which you live, This will help us to identify individual voices when we come to write up the interviews. This group discussion is to understand what would happen in your community if someone suffered an injury.

We would like to think particularly about four different aspects of what would happen, these aspects are seeking care (the time from an injury happens to taking action to get care), reaching care (the time until a facility or hospital that can treat the injury is reached), receiving quality care (getting good quality treatment for the injury at a facility or hospital), and remaining in care (for example, attending follow up at out-patients or going to rehabilitation).

**Seeking care**

Firstly, we wish to know what do people do straight after injuries occur?

Does the severity of the injury (how bad it is) determine what people do?

For people who need to go and get treatment, what happens after injuries?

What factors do you think make people more likely to seek health care?

What factors do you think make people less likely to seek health

care? Prompts, if necessary For example:

- Knowledge of availability of healthcare?

- Trust in healthcare?

- Fear of costs?

- Availability of a phone?

If people don’t seek healthcare what do they do instead?

**Reaching Care**

Now we wish to know what a person does after an injury has occurred and it has been decided

to seek health care.

Does what a person does depend on the severity of the injury?

What factors would make it more likely to reach a place of care quickly?

What factors make it less likely to reach a place of care

quickly?

Prompts, if necessary For example:

- Ambulance availability?

- Road conditions?

- Personal safety?

- Costs?

**Receiving care**

How about the care received after the injured person has made it to a place of

care?

Would you consider that this will be good quality care? Prompts, if

necessary

For example:

- Will the injured person get the correct treatment?

- Will they be treated with respect?

- Will they have to wait for a long time?

What factors do you think make it difficult for injured people to receive good quality

care?

What factors make it more likely they will receive good quality care? Prompts,

if necessary

For example:

- Qualifications of staff at the facility?

- Availability of equipment at the facility?- Personal contacts with staff at the facility?

- Costs of care?

**Remaining in care**

This is the final stage in the care-seeking process and we would like you to tell us about what

happens for people who are discharged from hospital or, when their injury has been mostly

treated, but, they have been asked to come back for more care, like out-patient appointments

or physiotherapy (rehabilitation)

Do most people who have been asked to return to (or remain in) care actually do so?

What are the barriers to people remaining in care?

What are the facilitators to people remaining in care?

Prompts, if necessary

For example:

- Distances needed to travel?

- Other family commitments?

Solutions

Finally, what do you think could be done to improve access to quality care for people who are

injured in your area?

Does anyone have any questions for us?

Thank you again for your time.

SWITCH THE DICTAPHONE OFF
