# Supplementary material for: Equitable access to quality trauma systems in Ghana: a qualitative study
Source: BMJ Open. 2025 Feb 12;15(2):e087095. doi: 10.1136/bmjopen-2024-087095 (PMC11822442; doi:10.1136/bmjopen-2024-087095)
Supplement: Supplementary file 2 [file bmjopen-2024-087095.reviewer_comments.pdf]

PEER REVIEW HISTORY

BMJ Open publishes all reviews undertaken for accepted manuscripts. Reviewers are asked to complete a checklist review form and are provided with free text boxes to elaborate on their assessment. These free text comments are reproduced below.

ARTICLE DETAILS

Title (Provisional)

EQUITABLE ACCESS TO QUALITY TRAUMA SYSTEMS IN GHANA: A QUALITATIVE STUDY

Authors

Kunfah, Sheba Mary Pognaa; Yakubu, Mustapha; Ofori-Appiah, Bernard; Ayingayure, Emmanuel; Odland, Maria Lisa; Yempabe, Tolgou; Buunaaim, Alexis Dun Bo-ib; Konadu-Yeboah, Dominic; Ignatowicz, Agnieszka; Davies, Justine; Stephen, Tabiri

---

VERSION 1 - REVIEW

Reviewer **1**

Name **Alayande, B**

Affiliation **University of Global Health Equity, Center for Equity in Global Surgery**

Date **28-Jun-2024**

COI **I have no competing interests, however, I contributed to the the same broader project, Equitrauma focusing my work on Rwanda, while the authors worked in Ghana.**

Thank you for this very important study identifying barriers to injury care in Ghana.

A few clarifications are necessary and some suggestions are included to improve the article.

1. The represented injuries appear to be skewed purposively towards RTAs, though the authors describe a deliberateness in recruiting patients with representative injuries. How were these injury types different? Can authors include detail on the specific type of injuries that these patients had? e.g. head injury, lower limb fractures, soft tissue injuries. This will give more clarity on selection.

2. With a gender lens, what was the premise of the selection of only 5 females for the individual interviews? How many of these were rural? Could this not have promoted under representation of females (specifically rural women) in this study?

3. Discussion- Authors should pivot from some absolute and total statements included in the text like “which is a wrong assessment of their injury”, “In our study the family head was the final decision maker concerning seeking care.” The results do not demonstrate these as total. Using terms like “are often”, “some respondents”, “suggests” does not straight away generalize the responses across urban and rural for every single participant. Page 9, lines 22, 32

4. Page 14, Table 1- Expand "others" using a footnote to the table, as readers would be curious to see this in more detail. The inclusion of bee stings as a component of injuries is interesting. It would be interesting and transparent to see other items included in the "others" category to facilitate critique.

5. Table 2, Page 15. For better and more consistent presentation, I would suggest to order the specific examples within the delays from most frequently mentioned to least frequently mentioned in each category. Delays 1 and 2 are ordered this way, but 3 and 4 are not. The consistency helps the reader.

6. Table 2, Page 15. While this is qualitative, including the percentage mention for each delay of the total mentions also helps. People understand percentages more than absolute figures.

7. A word cloud highlighting the various delays would have been very helpful to give the reader a visual of the various delays, and which ones were most cited.

8. Figure 1 could be removed. I would urge authors to challenge the relevance of this figure as it duplicates the information between urban and rural settings. I would argue that there is no benefit to this flowchart, as it just restates what is in the text and there is absolutely no difference between urban and rural causes of delay. In the results and the manuscript discussion, authors should outline whether there was any difference in the perceived barriers to injury care between rural and urban locations? Even if this is based on more commonly cited barriers by participants? If not, this figure should probably be removed as the content is in table 2.

Thank you

**Reviewer**	**2**
**Name**	**Haider, Adil**
**Affiliation**	**The Aga Khan University, Department of Surgery and Community Health Sciences**
**Date**	**28-Jun-2024**
**COI**	**none**

---

1) Although purposive sampling is mentioned as the methods to make sure that different injury types, ages, and genders are represented, the methodology section does not precisely outline the procedure followed in selecting participants. A more thorough explanataion would support the methods.

2) There is an imbalance in gender representation in the sample, with 39 men and 14 women. Since purposive sampling was designed to ensure fair representation, it would be useful to address the causes of this imbalance and describe how it may affect the study.

3) It shows that a high proportion of participants had been involved in road traffic crashes. It would be useful to clarify on how this may effect the generalizability of the study results to other injuries.

Thank you very much for clearly pointing out the limitations of the study. The quality and effect of your work would be further enhanced by making changes in light of the comments raised.

Reviewer 3

Name Nagasawa, Hiroki

Affiliation Juntendo Daigaku Igakubu Fuzoku Shizuoka Byoin, Department of Acute Critical Care Medicine

Date 30-Jul-2024

COI I have no competing interests to disclose.

I appreciate the opportunity to review your manuscript titled 'Equitable Access to Quality Trauma System in Ghana: A Qualitative Study.' The study aims to identify the barriers causing delays in accessing trauma healthcare services in Ghana.

I find this study well-structured and the manuscript well-written. I encourage the authors to expand on the results of this study to suggest improvements in public health quality for trauma care in Ghana or other regions facing similar issues.

Thank you for your attention to these matters.

Reviewer 4

Name Kettlewell, Jade

Affiliation University of Nottingham, Centre for Health Innovation, Leadership & Learning

Date 08-Aug-2024

COI None

Thank you for the opportunity to review this manuscript exploring the barriers to accessing quality trauma care after injury in Ghana. Whilst this is an important and interesting piece of work, there are a number of improvements that need to be made before it is suitable for publication.

I have added comments to the attached PDF, and have detailed my concerns and suggestions below.

Abstract:

- See comments about reporting numbers in the participant section.

- Detail missing from the design section about recording, transcription and analysis of data. Did you use a framework? Was it thematic analysis? Framework analysis? How many authors coded the data? Add the four-delay framework here.

Conclusion: Could be stronger, I feel like you have just repeated the four-delay framework again which isn't really concluding anything. What are the key takeaway messages from your work and what can this inform? What are your next steps? Future research?

Strengths and limitations: These could be more clear, for example: 'The use of both individual interviews and focus group discussions in this study brought to light the diverse lived experiences of patients' could be changed to 'The combination of individual interviews and focus groups effectively highlighted the diverse lived experiences of patients'.

Introduction:

- write numbers 10 and under in full, have pointed out in text on attached pdf - for example 'one in two'.

- The rationale for this study is really unclear and needs significant work. The whole introduction is very short and although you present some useful stats, I'm still not clear why this study needed conducting, what existing literature there is exploring access to quality care after trauma. Have any other studies explored access to trauma systems in other LMICs?

- You state 'However, the literature on injuries in Ghana usually focuses on prevention with little attention on access to quality healthcare after injury' - but where is that literature, it has not been cited or introduced?

- The four-delay framework is introduced at the end, but it might be helpful to briefly explain its relevance earlier in the introduction. Why was this framework chosen? Have other studies used it to explore trauma systems? How does it specifically apply to your context?

- While the introduction mentions that existing literature focuses on prevention rather than access to care post-injury, it could explicitly state the specific gap your study addresses. For example, how the lack of research on post-injury care impacts outcomes or policy decisions in Ghana?

- State your research question and aim more clearly, currently not sufficient.

- Also think about what you hoped this work would inform? Future research? Policy change? Identify issues to improve pathways?

Methods:

- Can you reference Equi-trauma? Website? Abstract?

- Page 3, line 41 - who was a 'key informant'? What was their background? Did they work in the hospital or were they a researcher? Make sure it is clear to an international audience.

- Not clear why you chose to conduct interviews and focus groups, and why only focus groups in some settings? Was it to encourage more participants in rural settings to participate?

- Were participants approached and invited to take part, given a participant information sheet and then decided if they wanted to take part? This information is missing. Did a research follow-up and recruit them?

Page 3, line 51 - did they provide written informed consent?

- Add the interview and focus group topic guides as an appendix and detail this in the text.

- Page 4 data analysis is well detailed and written. Just curious, did you use the framework approach to thematic analysis? Was it a deductive approach mapped on to the four-delay framework or did you used a deductive and inductive approach? see this ref https://bmcmedresmethodol.biomedcentral.com/articles/10.1186/1471-2288-13-117

- Did PPI have any involvement in data analysis/validation?

Results: really interesting findings and good quotes to illustrate the points you have made.

- Do you need to add FGD patient participant, did you assign these a number too? Would be better if each participant was assigned a study number like for the interview participants.

- the theme 'Delay in seeking care (delay 1)' mostly has quotes from those living in urban areas, was this a finding or do you have any more you can add from the rural setting?

- page 6, line 26 - what do you mean by Human Resources?

Discussion:

- I'd suggest starting with a stronger statement like 'this study is the first to explore barriers to accessing quality trauma care after injury in Ghana using the four-delay framework...'

- I think you could include parts of your discussion in your introduction section instead and then refer back to it here while comparing to your own results. You mention studies here that have not been introduced in the beginning of the manuscript even though they are relevant to your work (i.e. conducted in Ghana or exploring care pathways). The literature can be used to strengthen the introduction and the study rationale as suggested above.

- page 9, line 4 - write NHIS in full

- line 17 - 'This barrier caused patients to either default completely or partially' - not sure what you mean by this?

- good that you mention the implications of your findings here, this is not evidence in your abstract and would be good for you to add something to your introduction to talk about how your findings could inform policy change etc.

- could you be more specific about what future research should focus on, what are the next steps?

- suggest flipping your strengths and limitations around so strengths are first and limitations second

- ' The use of both individual interviews and focus group discussions in this study brought to light the diverse lived experiences of patients.- - think this could be more detailed and explain what the real value was of conducting both interviews and focus groups, what did it enable you to do? Were you able to recruit hard to reach communities? Would it have been hard to interview people from rural settings? Make it more clear why it is a strength as it is a common qualitative approach so not really a 'new' way of doing things.

Conclusion:

- Think this could be a lot stronger and really highlight what you found and how this could inform future research, policy change and help other similar LMICs improve services.

**** **The reviewer provided a marked copy with additional comments. Please contact the publisher for full details.** ****

Reviewer **5**

Name **Theologis, Tim**

Affiliation **Oxford University Hospitals NHS Foundation Trust, Paediatric Orthopaedic Surgery**

Date **13-Aug-2024**

COI **None**

This is a well written paper on an interesting subject. The paper presents a qualitative study on access to trauma care in Northern Ghana. The Authors used semi-structured interviews and focus group discussions followed by thematic analysis, using the four-delay framework.

To my knowledge, the subject of the study is original and I am not aware of any similar studies. I defer to qualitative research expertise on the methodology, which, from the clinical

perspective, appears adequate. The results are nicely presented and there are interesting quotations included, which highlight the problems that were identified.

The discussion would benefit from more work. In its current form, it repeats the results quite extensively, without a sufficiently in-depth analysis and critique. Critical appraisal of the existing literature would improve the discussion. Further discussion on the implications of the problems identified and potential ways of addressing them would also be helpful.

Overall, the paper is presented well, the text flows nicely and the illustrations are relevant and helpful.

VERSION 1 - AUTHOR RESPONSE

Reviewer: 1

Dr. B Alayande, University of Global Health Equity

S/N	COMMENT	RESPONSE	ADJUSTED SENTENCE IN THE MANUSCRIPT/PAGE NUMBER
5	1. The represented injuries appear to be skewed purposively towards RTAs, though the authors describe a deliberateness in recruiting patients with representative injuries. How were these injury types different? Can authors include detail on the specific type of injuries that these patients had? e.g. head injury, lower limb fractures, soft tissue injuries. This will give more clarity on selection.	The mechanisms of injuries apart from RTAs were bee stings and fall from a height. RTAs were the commonest forms of injuries found hence their over-representativeness in our study participants. Using injuries from mainly RTAs has been included as a limitation of this study.<br><br>The types of injuries were limb fractures, head injury, soft tissue injuries and dislocations	**(Page 12)**
6	With a gender lens, what was the premise of the selection of only 5 females for the individual interviews? How many of these were rural? Could this not have promoted under representation of females (specifically rural women) in this study?	The recruitment criteria was also based on participant availability and willingness during the data collection period. This also reflects the male predominance in prevalent injuries. However, the study acknowledges the potential underrepresentation of females, especially rural women, and recognizes this as a limitation in addressing the nuances of gendered experiences .	**(Page 12)**

		Of the 5 included females for the individual interviews, 3 were from the rural setting and 2 from the urban setting which, while offering some insight into rural female perspectives, may still not fully capture the gender-specific nuances.<br><br>This is a limitation we have acknowledged in the study	
7	Discussion- Authors should pivot from some absolute and total statements included in the text like “which is a wrong assessment of their injury”, “In our study the family head was the final decision maker concerning seeking care.” The results do not demonstrate these as total. Using terms like “are often”, “some respondents”, “suggests” does not straight away generalize the responses across urban and rural for every single participant. Page 9, lines 22, 32	Sentences have been adjusted.	“…which may be an incorrect clinical assessment of their injury.”<br>**(Page 10)**<br><br>“In our study the family head was likely the final decision maker concerning seeking care.”<br>(Page 10)
8	. Page 14, Table 1- Expand "others" using a footnote to the table, as readers would be curious to see this in more detail. The inclusion of bee stings as a component of injuries is interesting. It would be interesting and transparent to see other items included in the "others" category to facilitate critique.	Footnote has been added.	Others*- fall from heights, bee stings, blunt injury etc<br>Table and figures<br><br>**Table 1**
9	Table 2, Page 15. For better and more consistent presentation, I would suggest ordering the specific examples within the delays from most frequently mentioned to least frequently mentioned in each category. Delays 1 and 2 are ordered this way, but 3 and 4 are not. The consistency helps the reader.	Changes have been made. Delay 3 and 4 have also been ordered based on the most frequently measured to the least frequently measured	List of tables and figures<br><br>**Table 2**
10	Table 2, Page 15. While this is qualitative, including the percentage mention for each delay of the total	Percentages included as requested	**Table 2**

	mentions also helps. People understand percentages more than absolute figures.		
11	A word cloud highlighting the various delays would have been very helpful to give the reader a visual of the various delays, and which ones were most cited.	This has also been included	**Figure 1**
12	Figure 1 could be removed. I would urge authors to challenge the relevance of this figure as it duplicates the information between urban and rural settings. I would argue that there is no benefit to this flowchart, as it just restates what is in the text and there is absolutely no difference between urban and rural causes of delay. In the results and the manuscript discussion, authors should outline whether there was any difference in the perceived barriers to injury care between rural and urban locations? Even if this is based on more commonly cited barriers by participants? If not, this figure should probably be removed as the content is in table	The figure has been removed. The differences were in the number of times each delay was mentioned based on the settings. This has been mentioned in the manuscript	

Reviewer: 2
Dr. Adil Haider, The Aga Khan University

S/N	COMMENT	RESPONSE	ADJUSTED SENTENCE IN THE MANUSCRIPT/PAGE NUMBER
13	Although purposive sampling is mentioned as the methods to make sure that different injury types, ages, and genders are represented, the methodology section does not precisely outline the procedure followed in selecting participants. A more thorough explanation would support the methods.	The recruitment criteria was also based on participant availability and willingness during the data collection period. However, the study acknowledges the underrepresentation of females especially rural women and some injury types as a limitation in the study	**(Page 12)**
14	There is an imbalance in gender representation in the sample, with 39 men and 14 women. Since purposive sampling was designed to ensure fair representation, it would be useful to	Purposive and convenience sampling was used to recruit participants for the study. The recruitment	**(Page 12)**

	address the causes of this imbalance and describe how it may affect the study.	criteria was also based on participant availability and willingness during the data collection period. However, the study acknowledges the potential underrepresentation of females, especially rural women, and recognizes this as a limitation in addressing the nuances of gendered experiences. Adjustments have been made to the manuscript.	
15	It shows that a high proportion of participants had been involved in road traffic crashes. It would be useful to clarify on how this may effect the generalizability of the study results to other injuries.	This has been added as a limitation to the generalizability of the study.	"Similarly, most of those interviewed in this study had injuries from road traffic accidents and this affects the generalizability of the results."<br><br>**Page 12**, strengths and limitations

Reviewer: 3
Dr. Hiroki Nagasawa, Juntendo Daigaku Igakubu Fuzoku Shizuoka Byoin

S/N	COMMENT	RESPONSE	PARAGRAPH/PAGE NUMBER
16	I encourage the authors to expand on the results of this study to suggest improvements in public health quality for trauma care in Ghana or other regions facing similar issues.	This has been addressed as part of the conclusion of the study.	Conclusion, **Page 12**

Reviewer: 4

Dr. Jade Kettlewell, University of Nottingham

S/N	COMMENT	RESPONSE	ADJUSTED SENTENCE IN THE MANUSCRIPT/ PAGE NUMBER
17	See comments about reporting numbers in the participant section.<br>"don't need to write the number in brackets as well"<br>n=39 males, n=14 females, mean age =21 SD=15.6 etc.	Reporting numbers have been corrected.	**Participants**<br>Fifty-three patient participants (n=39 males, n=14 females, mean age =41, SD=15.6, n= 38 rural participants, n= 15 urban participants) who had an injury not more than 6 months preceding the start of the study.<br>**(Page 1)**<br><br>**Settings**<br>Fifteen individual interviews (n=15) and two focus group discussions (n=23) were conducted in Yendi (rural setting in Ghana) and ten individual interviews (n=10) and 1 focus group discussion (n=5) in the Tamale metropolis (urban setting in Ghana<br>**(Page 2)**
18	Detail missing from the design section about recording, transcription and analysis of data. Did you use a framework? Was it thematic analysis? Framework analysis?	Interviews and focus group discussions were audio recorded and transcribed. Two authors generated initial codes and four	Design, **Page 1**

	How many authors coded the data? Add the four-delay framework here.	authors cross validated them. Thematic analysis was done based the four-delay framework.	
19	Conclusion: Could be stronger, I feel like you have just repeated the four-delay framework again which isn't really concluding anything. What are the key takeaway messages from your work and what can this inform? What are your next steps? Future research?	The conclusion has been readdressed.	**(Page 12)**
20	Strengths and limitations: These could be more clear, for example: 'The use of both individual interviews and focus group discussions in this study brought to light the diverse<br>lived experiences of patients' could be changed to 'The combination of individual interviews and focus groups effectively highlighted the diverse lived experiences of patients'.	The strengths and limitation section has been rewritten, and the stated portion has been addressed.	The combination of individual interviews and focus groups was to enhance the depth, breadth, and validity of our study for a richer and more nuanced understanding of the topic.<br>**(Page 12)**
21	The rationale for this study is really unclear and needs significant work. The whole introduction is very short and although you present some useful stats, I'm still not clear why this study needed conducting, what existing literature there is exploring access to quality care after trauma. Have any other studies explored access to trauma systems in other LMICs?	The entire introduction has been rewritten to include the suggestions mentioned	**(Page 3-4)**
22	You state 'However, the literature on injuries in Ghana usually focuses on prevention with little attention on access to quality healthcare after injury' - but where is that literature, it has not been cited or introduced?	The entire introduction has been rewritten to include the suggestions mentioned	**(Page 3-4)**
23	- The four-delay framework is introduced at the end, but it might be helpful to briefly explain its relevance earlier in the introduction. Why was this framework chosen? Have other studies used it to explore trauma systems? How does it specifically apply to your context?	The entire introduction has been rewritten to include the suggestions mentioned	**(Page 3-4)**

24	- While the introduction mentions that existing literature focuses on prevention rather than access to care post-injury, it could explicitly state the specific gap your study addresses. For example, how the lack of research on post-injury care impacts outcomes or policy decisions in Ghana?	The entire introduction has been rewritten to include the suggestions mentioned	**(Page 3-4)**
25	- State your research question and aim more clearly, currently not sufficient.<br>- Also think about what you hoped this work would inform? Future research? Policy change? Identify issues to improve pathways?	The entire introduction has been rewritten to include the suggestions mentioned	**(Page 3-4)**
26	- Can you reference Equi-trauma? Website? Abstract?	The entire introduction has been rewritten to include the suggestions mentioned	**(Page 3-4)**
27	Page 3, line 41 - who was a 'key informant'? What was their background? Did they work in the hospital or were they a researcher? Make sure it is clear to an international audience.	The term 'key informant' has been changed.	"In the urban setting, a community member who was familiar with the community was recruited..."<br><br>**(Page 4)**
28	- Not clear why you chose to conduct interviews and focus groups, and why only focus groups in some settings? Was it to encourage more participants in rural settings to participate?	There were interviews and focus group discussions in both the rural and urban settings however there were 2 focus group discussions conducted in the rural setting and 1 in the urban setting as shown in table 1. The focus group discussions in the rural setting were carried out in two separate rural communities which were geographically and culturally distinct hence we had more focus group discussions from the rural area.	Results, **Page 6**
29	- Were participants approached and invited to take part, given a participant information sheet and then decided if they wanted to take part? This information is missing. Did a research follow-up and recruit them? Page 3, line 51 - did they provide written	This information has been added	: "In the urban setting, a community member who was familiar with the community was recruited to assist two trained

	informed consent?		Research Assistants to identify eligible participants . In the rural setting, health workers in Health Centers reviewed patient records, to identify the eligible participants together with the Research Assistants. Participants were contacted on phone and with the assistance of a recruited community member, they were traced to their homes to share information about the study using participant information sheets. After about 1-2 weeks these participants were contacted to assess their willingness to be part of the study and to book a convenient location for the data collection. If they were willing to be part of the study, they were asked to sign or thumb print a consent form before data collection took place.”<br><br>**(Page 4)**
30	- Add the interview and focus group topic guides as an appendix and detail this in the text.	These have been added to the supplementary documents	**(page 5)**

	Page 4 data analysis is well detailed and written. Just curious, did you use the framework approach to thematic analysis? Was it a deductive approach mapped on to the four-delay framework or did you used a deductive and inductive approach? see this ref https://bmcmedresmethodol.biomedcentral.com/articles/10.1186/1471-2288-13-117	We did not use the framework approach. We did a thematic analysis Codes were generated inductively (from data) and inductively based on the four-delay framework.	
31	Did PPI have any involvement in data analysis/validation?	They were involved in the recruitment processes and the validation of the data.	
32	Results: really interesting findings and good quotes to illustrate the points you have made.	Thank you	**Page 6**
33	Do you need to add FGD patient participant, did you assign these a number too? Would be better if each participant was assigned a study number like for the interview participants.	Patient participants for focus group discussions were assigned numbers and this has been included in the study.	**Pages 6-9**
34	- the theme 'Delay in seeking care (delay 1)' mostly has quotes from those living in urban areas, was this a finding or do you have any more you can add from the rural setting?	Another quote has been added from the rural setting under this delay	**Page 6**
35	- - page 6, line 26 - what do you mean by Human Resources?	The statement has been changed	"…understaffing of health facilities"<br>**Page 8**
	Discussion:<br>- I'd suggest starting with a stronger statement like 'this study is the first to explore barriers to accessing quality trauma care after injury in Ghana using the four-delay framework...'<br>- .<br>- page 9, line 4 - write NHIS in full	The necessary changes have been made to the manuscript.<br>NHIS has been written in full	National Health Insurance Scheme.<br>**(Page 11)**
36	I think you could include parts of your discussion in your introduction section instead and then refer back to it here while comparing to your own results. You mention studies here that have not been introduced in the beginning of the manuscript even though they are relevant	The introduction has been rewritten to address these	**(Page 3-4)**

	to your work (i.e. conducted in Ghana or exploring care pathways). The literature can be used to strengthen the introduction and the study rationale as suggested above		
37	line 17 - 'This barrier caused patients to either default completely or partially' - not sure what you mean by this?	The sentence has been changed.	“Hence, preventing injured patients from complying with their follow-up plans such as for physiotherapy”<br>**(Page 11)**
38	good that you mention the implications of your findings here, this is not evidence in your abstract and would be good for you to add something to your introduction to talk about how your findings could inform policy change etc.	This has been included in the conclusion of the abstract.	**Page 2**
39	- could you be more specific about what future research should focus on, what are the next steps?<br>- suggest flipping your strengths and limitations around so strengths are first and limitations second	The implications for future research has been added to the conclusion. The strengths and limitations have been flipped over.	**Page 12**
40	- ' The use of both individual interviews and focus group discussions in this study brought to light the diverse lived experiences of patients.- - think this could be more detailed and explain what the real value was of conducting both interviews and focus groups, what did it enable you to do? Were you able to recruit hard to reach communities? Would it have been hard to interview people from rural settings? Make it more clear why it is a strength as it is a common qualitative approach so not really a 'new' way of doing things.	This has been addressed in the strengths and limitations portion of the manuscript.	**Page 12**
41	Conclusion:<br>Think this could be a lot stronger and really highlight what you found and how this could inform future research, policy change and help other similar LMICs improve services.	The conclusion has been rewritten to include all the suggested components.	**Page 12**

Reviewer: 5
Prof. Tim Theologis, Oxford University Hospitals NHS Foundation Trust
Comments to the Author:

S/N	COMMENT	RESPONSE	ADJUSTED SENTENCE IN THE MANUSCRIPT/PAGE NUMBER
42	The discussion would benefit from more work. In its current form, it repeats the results quite extensively, without a sufficiently in-depth analysis and critique. Critical appraisal of the existing literature would improve the discussion. Further discussion on the implications of the problems identified and potential ways of addressing them would also be helpful.	Some additions have been made to the discussion to improve it	**Pages 10-11**

VERSION 2 - REVIEW

Reviewer 1
Name Alayande, B
Affiliation University of Global Health Equity, Center for Equity in Global Surgery
Date 27-Dec-2024
COI

Thank you. All reviewer comments have been addressed.
